# Epidemiology of orthodontic treatment need in southwestern Ethiopian children: a cross sectional study using the index of orthodontic treatment need

**DOI:** 10.1186/s12903-020-01196-2

**Published:** 2020-07-22

**Authors:** Mulualem Tolessa, Amit T. Singel, Hailu Merga

**Affiliations:** 1 Department of Dentistry, Institute of Health, Jimma University, Jimma, Ethiopia; 2 Department of Epidemiology, Institute of Health, Jimma University, Jimma, Ethiopia

**Keywords:** Epidemiology, Orthodontic treatment need, Children, Ethiopia

## Abstract

**Background:**

The planning of orthodontic treatment within a public health system requires information on the orthodontic treatment needs of the population. It is important to have epidemiological data to estimate the total need for orthodontic care in any region. The present study aimed to determine the orthodontic treatment need in 12 years old Southwestern Ethiopian children.

**Methods:**

The institution-based cross-sectional study was conducted which involved 347 twelve years old school children randomly selected from seven different public schools in Jimma Town, Southwestern Ethiopia. A structured interview and clinical examination were used to assess the subjects. One examiner used Dental Health Component (DHC) and Aesthetic Component (AC) of the Index of Orthodontic Treatment Need (IOTN) to estimate the treatment need. Descriptive statistics and chi-square tests were used for data analysis with statistical significance set at *P* < 0.05.

**Results:**

According to the DHC of IOTN, almost half of the subjects in the sample were in moderate to the great need for orthodontic treatment. About 15% of the children had a great need for orthodontic treatment based on IOTN-AC. The most prevalent occlusal traits for defining the DHC categorization include increased Overjet (30.8%) and Crowding (23.3%). There was no statistical difference in the distribution of DHC grades and AC scored based on gender.

**Conclusion:**

This study revealed that the need for orthodontic treatment was high. The percentage of the need for orthodontic treatment is higher in comparison to most of the studies conducted in African regions. Therefore, publicly subsidized orthodontic treatment should be planned and provided to those who are in great need for orthodontic treatment. Besides, awareness about orthodontic treatment should also be considered.

## Background

Malocclusion is an appreciable deviation from an ideal occlusion [1]. Many of these deviations are within the range of what is to be considered as normal biologic variation. However, some deviations may have negative influence on dento-facial development, contributing to impaired oral functions, susceptibility to facial traumatic injuries and development of caries and periodontal problems [2, 3]. In addition, malocclusion could cause psychosocial problems related to impaired/altered dento-facial esthetics [2, 4, 5]. Orthodontic treatments comprise a large proportion of dental treatment and in most cases they are carried out during adolescence and early adulthood to solve malocclusion problems [2].

The planning of orthodontic treatment within a public health system requires information on the orthodontic treatment needs of the population [6, 7]. With the growing demand for orthodontic treatment, a variety of clinician based indices have been developed to classify various types of malocclusion, and determine their orthodontic treatment need [3, 8]. These indices can be used in estimating orthodontic treatment need, prioritizing treatment need in patients referred for orthodontics particularly where there are limited resources for orthodontics among pubic health care services, and safeguarding for the patients [8, 9]. One of the most commonly used indices that assess the orthodontic treatment needs among children and adults is the Index of Orthodontic Treatment Need (IOTN), which was developed by Brook and Shaw. The IOTN has two separate components, the aesthetic (AC) and dental health components (DHC), which rank malocclusion in increasing priority according to aesthetic considerations and dental health implication [10].

Various studies have used the index of orthodontic treatment need (IOTN) for measuring the degree of malocclusion and the need for orthodontic treatment in different population sectors. For instance, the prevalence of orthodontic treatment need using IOTN-DHC was 21.3% in France [11], 22% in Tanzania [12], 28% in Kuwait [13], 34.2% in Brazil [14], 34% in Jordan [15], 36.1% in Iran [16], 38.8% in Turkey [17], and 71.6% in Saudi Arabia [18].

With the improvement of the socio-economic situation in Ethiopia, the demand for orthodontic treatment is increasing quite rapidly. Many patients with malocclusion problems visit dental clinics in both government and private health facilities. However, orthodontic concern like other oral health care procedures is given low priority in the health care system because of the high cost of treatment and the shortage of orthodontists. In the light of the above reasons, and since no previous study on Ethiopian children has been conducted, it is important to have epidemiological data to estimate the total need for orthodontic care in this region. The objective of this study was therefore to determine the need for orthodontic treatment using the Index of Orthodontic treatment need and its gender distribution in 12-year-old schoolchildren belonging to Jimma Town, located in southwestern Ethiopia.

## Methods

### Study design and setting

An institution-based cross-sectional study was conducted from October 20–November 4, 2018 among 12 years old schoolchildren in Jimma town, with no history of orthodontic treatment. Jimma Town is located approximately 350 km southwest of the capital Addis Ababa. In the town, there were 43 public schools, out of which 22 were public and 21 private primary schools. A total number of 12 years old school children attending public primary schools were 3474.

### Sample size and population

All randomly selected 12 year old school children from selected public primary schools of Jimma town during the study period were the study population for the study. Accordingly, the sample size was calculated using single population proportion formula with the following assumptions: 50% proportion of orthodontic treatment need (using DHC of IOTN) among 12 years old school children, a maximum tolerable error of 5% and a 95% confidence interval. Since the source population was less than 10,000, a population correction factor was employed and after adding a 10% non-response rate the final sample size became 346 children. To get the subjects, first 7 schools (30%) were randomly selected from the available 22 public primary schools. Then, samples were proportionally allocated and computed from each selected school with their corresponding population size. Finally, sampling frames of the study subjects were obtained from the list of 12 years old school children from each selected primary school and a simple random sampling technique (lottery method) was used to get study subjects from each selected school.

### Data collection and measurement

Data was collected using a structured interviewer-administered questionnaire which was developed after reviewing different literature (S1) and clinical examination. The data collection tool included socio-demographic characteristics, Dental Health Component of IOTN and Aesthetic Component of IOTN. A structured face-to-face interview was carried out before the respective clinical examination of each child. The children/caregiver answered questions related to socio-demographic characteristics. Thereafter, one examiner conducted the clinical examination and rated the children AC scores. The examination was carried out in natural light using latex gloves, mouth mirror, and digital caliper. No radiographs and study casts were used. All occlusal anomalies of the DHC were recorded and scored separately in an individual form. The DHC grade was then determined according to the highest-scoring anomaly. To examine the AC, a cheek retractor was applied and the appearance of the teeth was compared to the AC photographs.

The questionnaire used to collect data was prepared in English version and translated into local languages, Afan Oromo and Amharic, and back to English to check the consistency. To assure the quality of data, the examiner was trained and calibrated in the use of the IOTN before data collection began. He was evaluated on a set of 20 plaster casts previously examined (gold standard). To test his (intra-examiner) reliability, these casts were re-examined after 7 days, and evaluated by the Kappa statistics. The intra-examiner agreement for DHC showed a Kappa value of 0.914 and for AC showed 0.821, indicating ‘high agreement’ between the first and second readings. Training was also given to data collectors (dental interns) to orient them on the objectives, sampling procedures, how to approach the study subjects, and the ethical conduct of the study. Everyday case sheets were reviewed to ensure the accuracy of each data.

### Statistical analysis

STROBE checklist was used to analyze and report data [19]. Data was cleaned, checked for missing values, entered into EpiData version 3.1, and analyzed using SPSS (IBM SPSS Statistics for Macintosh, Version 20.0. Armonk, NY). Descriptive statistics were calculated for the IOTN DHC grades and IOTN AC scores. The IOTN results were analyzed with regard to gender using the chi-square test. Differences greater than (*P* < 0.05) were considered statistically significant.

## Results

A total of 347 aged 12 years old school children participated in the study, giving a response rate of 91.08%. In this study, more than half of the children under study were girls and none of them had a history of orthodontic treatment. About two-thirds (63.4%) of these children were Oromo by their ethnicity and more than half (54.5%) of them were Muslims by their religion. The majority of them (48.4%) were from grade 5 (Table 1).

**Table 1:** Socio-demographic characteristics of the school children for the assessment of Orthodontic treatment need in southwestern Ethiopian, 2018

Variables	Category	Frequency (Percentage) N (%)
Gender	Male	155 (44.7)
	Female	192(55.3)
Ethnicity	Oromo	220(63.4)
	Amhara	42(12.1)
	Dawro	34(9.8)
	Others	51(14.7)
Religion	Muslim	189(54.5)
	Orthodox	112(32.3)
	Others	46(13.3)
Grade	Grade 1–4	84(24.2)
	Grade 5	168(48.4)
	Grade 6	95(27.4)

### Orthodontic treatment need, IOTN-DHC

An objective treatment need was recorded in 30% of the schoolchildren; 18.1%assigned to borderline need and 51.9% to little/no need for orthodontic treatment. No statistical difference with regard to DHC grades was found between genders (χ 2 = 0.668, *P* > 0.05) (Table 2).

**Table 2:** Frequency of the dental health component of the Index of Orthodontic Treatment Need by gender among school children in southwestern Ethiopia, 2018

IOTN (DHC) Category	Grades	Gender		Total	*p*-value	χ2
		Male, N (%)	Female, N (%)			
No/Little need	1–2	84 (54.2%)	96 (50%)	180(51.9%)	0.716	0.668
Borderline need	3	26 (16.8%)	37 (19.3%)	63 (18.2%)		
Definite need	4–5	45 (29%)	59 (30.7%)	104 (30%)		
Total		155 (100%)	192 (100%)	347		

### Orthodontic treatment need, IOTN-AC

Orthodontic treatment needs according to aesthetic impairment is shown in Table 3. In 61.7% of children, the treatment need was either slight or not indicated, 23.1% had a borderline need, while 15.3% were considered to have a definite treatment need. There was no statistical gender difference in the IOTN AC scores (χ 2 = 2.617; *P* > 0.05) (Table 3).

**Table 3:** Frequency of the Aesthetic component of the Index of treatment need by gender among school children in southwestern Ethiopia, 2018

IOTN (AC) Category	Grade	Gender		Total	*p*-value	X2
		Male N (%)	Female N (%)			
No/little need	1–4	98 (63.2%)	116 (60.4%)	214 (61.7%)	0.270	2.617
Borderline need	5–7	30 (19.4%)	50 (26.0%)	80 (23.1%)		
Definite need	8–10	27 (17.4%)	26 (13.5%)	53 (15.3%)		
Total		155 (100%)	192 (100%)	347		

### DHC Occlusal features

The occlusal features found in the children according to DHC scores are shown under Table 4.

**Table 4:** Distribution of occlusal traits according to the level of orthodontic treatment need (DHC) among school children in southwestern Ethiopia, 2018

Malocclusion	Total		Treatment need – IOTN (DHC)					
			No/Little		Moderate		Definite	
	N(%)		N (%)		N (%)		N (%)	
Increased overjet (a)	107(30.8)		49(14.1)		32(9.2)		26(7.5)	
Reverse overjet (b, m)	3(0.9)		0		0		3(0.9)	
Contact point displacement (d)	81(23.3)		35(10.0)		26(7.5)		20(5.8)	
Open bite (e)	5	1.4	2	0.6	3	0.9		
Increased overbite (f)	6	1.7	4	1.1	2	0.6		
Crossbite (c, l)	17	4.9	13	3.7	1	0.3	3	0.9
Tooth absence (h)	13	3.8					13	3.8
Supernumerary teeth (x)	4	1.2					4	1.2
Partially erupted, tipped or impacted teeth (t)	17	4.9					17	4.9
Impeded eruption of teeth (i)	18	5.2					18	5.2
Prenormal or postnormal occlusions	29	8.4	29	8.4				
Normal occlusion or Minor malocclusions	47	13.5	47	13.5				

The most frequent occlusal traits for the group of definite need for orthodontic treatment were the following: increased Overjet (6 mm or greater) (7.5%), contact point displacement of greater than 4-mm (crowding) (5.8%), impeded eruption of teeth (5.2%), and partially tipped, erupted or impacted teeth (4.9%) (Table 4).

## Discussion

The results of objective need for orthodontic treatment in this study provide baseline data for planning orthodontic services in Ethiopia. The need for orthodontic treatment based on the DHC scores showed that almost half of the children (48.2%) were in need for orthodontic treatment when the subjects with the moderate or great need for treatment were summed up. A definite need for orthodontic treatment need according to DHC was observed in 30% of the study subjects. This finding is similar to the findings from UK, 32.7% [10], Kuwait, 28% [13], New Zealand, 31.3% [20], Italy, 27.3% [21] and Peru 29.9% [22]. However, the percentage for definite treatment need in this study is higher than the study from Tanzanian schoolchildren [12]. It is also higher than the 18.1% reported for 12-year-old Sahrawi children [23],21% of Saudi Arabian adolescents [24], 21.3% of the 9–12-year-old French schoolchildren [11], 24.7% of 11–15-year-old Bangladesh school children [25] and 15.3% for 12 years old Romanian schoolchildren [26]. On the other hand, a study conducted on 250 school children aged 11–14 years, and 250 patients aged 11–14 years in Turkey showed that 38.8% of the school population and 83.2% of the referred population needed definite orthodontic treatment need [17]. Other studies have also reported a higher finding than the present study [15, 16, 18, 27]. The reported differences in normative orthodontic treatment need may be due to the different methods used and differences among the age groups studied. In addition, some studies included samples with a history of orthodontic treatment and referred for orthodontic treatment.

In the IOTN-DHC index, only the most severe occlusal trait is considered for categorization, despite the fact that other severe symptoms may be present. In this study, the two most common occlusal traits responsible for the final DHC categorization were increased overjet and contact point displacement. Similarly, some previous studies reported increased overjet as the most common trait, followed by crowding [28, 29]. Other studies have found contact point displacement as the most common occlusal trait followed by increased overjet [11, 22, 30]. These observations have public dental health implications because increased overjet and crowding are the occlusal traits commonly associated with traumatic injuries and poor periodontal conditions [31]. Studies also show that increased overjet and inadequate lip coverage are important contributing factors for traumatic dental injuries and that reducing a large overjet is not only beneficial from an aesthetic standpoint but also minimizes the risk of trauma and long-term complications to the dentition [30, 32].

Professional assessment of orthodontic treatment need according to AC of the IOTN, classified 15.3% of the children as being in definite need for orthodontic treatment. This figure is comparable to 13.7% of the 12 years old Western Saharan children [23] and 11.4% of the 12 years old Romanian schoolchildren [26]. In contrast, a study from Brazil found that 8.1% of the 12 years old school children had definite treatment need according to AC of IOTN, which was lower than the current study. In their study, the authors didn’t found a significant gender difference in dental appearance perception corroborating the present findings [28]. Aesthetic Component of IOTN, whether it is examiner or patient-based, assesses malocclusion on the grounds of aesthetic impairment, and by inference reflects the psycho-social need for orthodontic treatment. However, it has shortcomings like the other esthetic orthodontic treatment need indices; the subjective nature of the aesthetic indices and the variable perception of attractiveness between clinicians and patients, and among various cultures or countries [33].

The present study has also found high discrepancy in treatment needs between the DHC and AC of IOTN. The discrepancy may be attributed to the fact that malocclusion traits like missing teeth, crossbites, deep traumatic overbites, non-erupted or impacted teeth has definite need for orthodontic treatment) have dental health implications, but do not attract a high Aesthetic Component score. In addition, as AC is subjective in its nature and assesses the aesthetic aspects of the malocclusion only in frontal view, it brings difficulties in assessing some parameters, such as degrees of Overjet and Overbite [8]. Hence, these indices show different aspects of orthodontic treatment need, both of which can be used to complement each other in epidemiologic surveys and diagnostic procedures.

Dental Health Component of IOTN is a valuable tool in determining the treatment need priority for effective resource use in orthodontic care. It is based on the view that the more a deviation differs from a given norm (the ideal occlusion), the greater are the risks of future objective functional deficits or oral health problems. However, scientific evidence to assess its validity is lacking [34, 35]. Due to this limitation of DHC of IOTN, a recently conducted path model study suggested that orthodontic treatment need assessments should be based on the consequences of malocclusion for the individual [36]. The results of the present study are useful for public health planning and serve as a baseline data for future studies.

The limitations of this study were, since it is institution-based study its generalization will be limited to similar institutions only. Further, this study was performed in a specific age group, which could affect the generalizability of the findings. On the other hand, the possibility of interviewer bias during interviews. Besides, inter-examiner reliability was not tested because we had only one examiner who carried out the clinical examination.

## Conclusion

The present study has found that a high percentage of the 12 years old school children were in need of orthodontic treatment need. Increased overjet and Crowding were the most common occlusal features defining the DHC categorization. There was no statistical difference with regard to DHC grades and AC scores between genders. Therefore, publicly subsidized orthodontic treatment should be provided to those who are in great need for orthodontic treatment. Moreover, cost-benefit and cost-effectiveness analyses should be carried out to assess the acceptability of the level of service. The study may also serve as a baseline study for future studies.

## Supplementary information

**Additional file 1: S1.** Tool for the investigation of Epidemiology of Orthodontic treatment need.

## Data Availability

The datasets used and/or analysed during the current study available from the corresponding author on reasonable request.

## Abbreviations

AC: Aesthetic Component
DHC: Dental Health Component
IOTN: Index of Orthodontic Treatment Need
SPSS: Statistical Package for Social Sciences
