# Supplementary material for: Epidemiology of orthodontic treatment need in southwestern Ethiopian children: a cross sectional study using the index of orthodontic treatment need
Source: BMC Oral Health. 2020 Jul 22;20:210. doi: 10.1186/s12903-020-01196-2 (PMC7376854; doi:10.1186/s12903-020-01196-2)

nTools: Questionnaire

This questionnaire is part of a research thesis being conducted by Dr Mulualem Tolessa. All information you give is confidential.

Do you want to be part of the study?

Yes No

| Part One: - Socio-demographic Information | | |  |
| --- | --- | --- | --- |
| Q.# | Question | Codes ­­­­________ |  |
| 101 | Gender | 1. Male ___________  2. Female __________ |  |
| 102 | What is your Ethnicity? | 1. Oromo _______  2. Amhara ______  3. Dawro _______  4. Tigre _______  5. Kefa _______  6. Other (specify) ___________ |  |
| 103 | What is your religion? | 1.Orthodox _______  2.Islam _______  3.Protestant _______  4.Catholic _______  5.Other (specify) ____________ |  |
| 104 | What grade are you attending? | _________ grade |  |
| Part two:- Normative Need based on Dental Health Component. Mark **(X)** on the observed trait | | | |
| 105 | CLP | 5p: defects of cleft lip and palate and other craniofacial anomalies | ____ |
| 106 | Missing teeth | 5h: Extensive hypodontia with restorative implications (more than one tooth per quadrant) requiring pre-prosthetic orthodontics | ____ |
|  |  | 5i: Impeded eruption of teeth (except third molars) due to crowding displacement, the presence of supernumerary teeth, retained deciduous teeth, and any pathological cause | ____ |
|  |  | 5s: Submerged deciduous teeth | ____ |
|  |  | 4h: Less extensive hypodontia requiring pre restorative orthodontics or orthodontic space closure (one tooth per quadrant) | ____ |
| 107 | Increased Overjet | 5a: Increased overjet > 9 mm | ____ |
|  |  | 4a: Increased overjet > 6mm but =<9 mm | ____ |
|  |  | 3a: Increased overjet > 3.5 mm but =<6 mm with incompetent lips | ____ |
|  |  | 2a: Increased overjet >3.5 mm but =< 6 mm with competent lips | ____ |
| 108 | Reverse Overjet | 5m: Reverse overjet >3.5 mm with reported masticatory and speech difficulties | ____ |
|  |  | 4m: Reverse overjet > 1 mm but < 3.5 mm with recorded masticatory or speech difficulties | ____ |
|  |  | 4b: Reverse overjet > 3.5 mm with no masticatory or speech difficulties | ____ |
|  |  | 3b: Reverse overjet > 1 mm but =< 3.5 mm | ____ |
|  |  | 2b: Reverse overjet >0 mm but =<1 mm | ____ |
| 109 | Crossbite | 4l: Posterior lingual cross bite with no functional occlusal contact in one or both buccal segments | ____ |
|  |  | 4c: Anterior or posterior crossbites with > 2mm discrepancies between retruded contact position and intercuspal position | ____ |
|  |  | 3c: Anterior or posterior crossbites with >1 mm but = < 2 mm discrepancies between retruded contact position and intercuspal position | ____ |
|  |  | 2c: Anterior or posterior cross bite with =< 1 mm discrepancy between retruded contact position and intercuspal position | ____ |
| 110 | Displacement of contact point | 4x: Presence of supernumerary teeth | ____ |
|  |  | 4t: Partially erupted teeth, tipped, and impacted against adjacent teeth | ____ |
|  |  | 4d: Severe contact point displacements >4 mm | ____ |
|  |  | 3d: contact point displacement> 2 mm but =< 4 mm | ____ |
|  |  | 2d: Contact point displacement > 1 mm but =< 2 mm | ____ |
| 111 | Overbites | 4e: Extreme lateral or anterior open bites > 4mm. | ____ |
|  |  | 3e: Lateral or anterior open bite > 2 mm but =< 4mm | ____ |
|  |  | 2e: Anterior or posterior open bite > 1 mm but =< 2 mm | ____ |
| 112 | Deepbite | 4f: Increased and complete overbite with gingival or palatal trauma | ____ |
|  |  | 3f: Deep Overbite complete on gingival or palatal tissues but no trauma | ____ |
|  |  | 2f: Increased overbite >= 3.5 mm without gingival contact | ____ |
| 113 | Molar relationship | 2g: Pre-normal or post-normal occlusions with no other anomalies | ____ |
| 114 |  | 1. Extremely minor malocclusions including contact point displacements < 1 mm | _____ |

Part 3: Looking at the pictures below, choose what number picture do you think looks most like to the child’s teeth? _______________


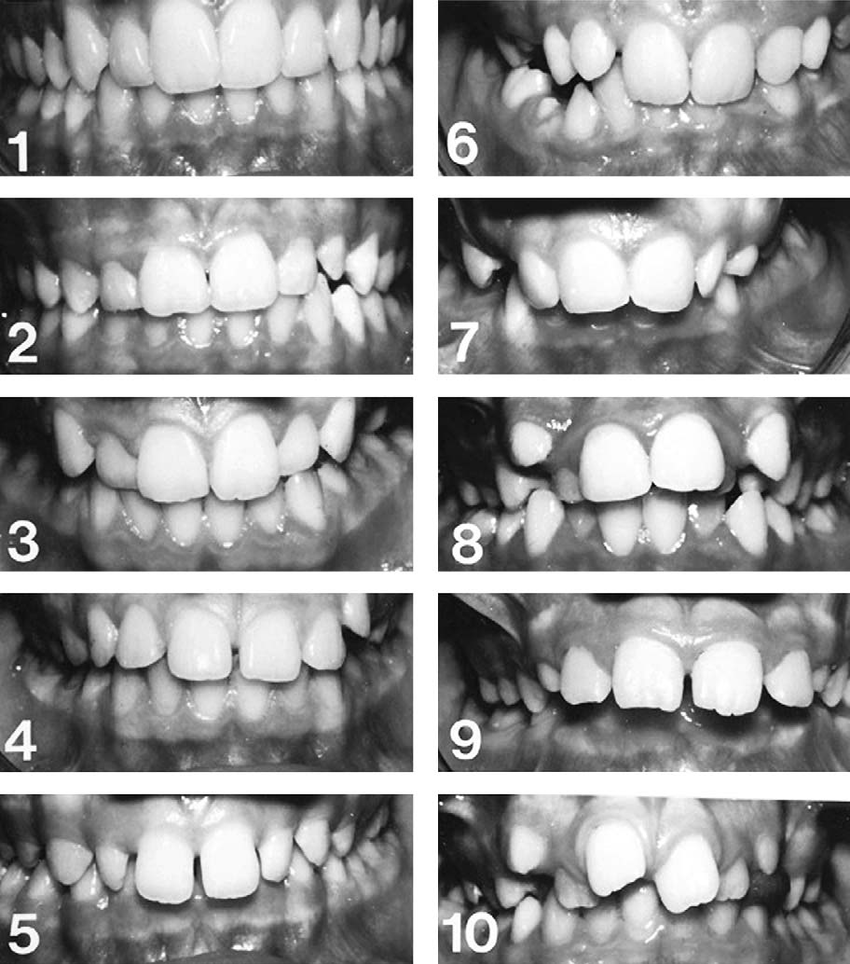

Supplement: Supplementary file 1 — Additional file 1: S1. Tool for the investigation of Epidemiology of Orthodontic treatment need. [file 12903_2020_1196_MOESM1_ESM.docx]
